# Effect of crown retention systems and loading direction on the stress magnitude of posterior implant-supported restorations: A 3D-FEA

**DOI:** 10.1016/j.heliyon.2024.e28129

**Published:** 2024-03-14

**Authors:** João Paulo M. Tribst, Niek de Jager, Amanda M.O. Dal Piva, Cees J. Kleverlaan, Albert Feilzer

**Affiliations:** a Academic Centre for Dentistry Amsterdam (ACTA), Department of Department of Reconstructive Oral Care, Universiteit van Amsterdam en Vrije Universiteit Amsterdam, 1081, LA, Amsterdam, the Netherlands; b Academic Centre for Dentistry Amsterdam (ACTA), Department of Dental Materials, Universiteit van Amsterdam en Vrije Universiteit Amsterdam, 1081, LA, Amsterdam, the Netherlands

**Keywords:** Crowns, Dental abutments, Dental implants, Finite element analysis, Prosthodontics

## Abstract

This study aimed to investigate the effect of four retention systems for implant-supported posterior crowns under compressive loading using three-dimensional finite element analysis. A morse-taper dental implant (4.1 × 10 mm) was designed with Computer Aided Design software based on non-uniform rational B-spline surfaces. According to International Organization for Standardization 14,801:2016, the implant was positioned at 3 mm above the crestal level. Then four models were designed with different crown retention systems: screw-retained (A), cement-retained (B), lateral-screw-retained (C), and modified lateral-screw-retained (D). The models were imported to the analysis software and mesh was generated based on the coincident nodes between the juxtaposed lines. For the boundary conditions, two loads (600 N) were applied (axial to the implant fixture and oblique at 30°) totaling 8 conditions according to retention design and loading. The von-Mises stress analysis showed that different retention systems modify the stress magnitude in the implant-supported posterior crown. There is a similar stress pattern in the implant threads. However, models C and D presented higher stress concentrations in the crown margin in comparison with A and B. The oblique loading highly increased the stress magnitude for all models. In the simulated conditions, part of the stress was concentrated at the lateral screw under axial loading for model C and oblique loading for model D. The results indicate a possible new failure origin for crown retained using lateral screws in comparison to conventional cement-retained or screw-retained systems.

## Highlights

• Stress pattern is affected by retention systems on implant-supported crowns.
• Higher stress concentrations in crown margin for lateral-screw designs.
• Potential new failure origin for lateral-screw-retained crowns.
• Oblique loading significantly increases stress magnitude.

## Abbreviation list

3D: Three-dimensional
FEA: Finite element analysis
CAD: Computer-aided design
ISO: International Organization for Standardization
STEP: Standard for the Exchange of Product Data
3YTZP: 3 mol% yttria-stabilized tetragonal zirconia polycrystal

## 1 Introduction

The high success rate of dental implants can be directly associated with aesthetics and mechanical conditions after prosthetics rehabilitation [1]. Despite dental implants having a high success rate, failures can occur which compromises the dental treatment longevity, especially in unitary implant-supported restorations with overload conditions [[2], [3], [4]].

Considering the implant-supported prosthesis design, the literature presents several factors related to it. One of these factors is the crown retention system during the prostheses manufacturing, which can be didactically divided into cement or screw-retained [[5], [6], [7]]. However, there is no consensus regarding the most suitable type of prosthesis retention for the implant treatment's long-term success, reflecting only the professional's personal choice for the type of retention that will be used in each clinical case [8]. Despite that, each type of prosthesis design presents singularities: the screw-retained can be easily removed when maintenance is needed; however, the screw-access hole is a required feature that can impair the esthetics [[5], [6], [7], [8]]. In addition, the screw-access hole must be sealed with a resin composite that can be more worn than the crown restorative material or can be even lost, compromising the occlusal surface anatomy [9]. Although the absence of a screw-access hole in cement-retained design can improve the crown esthetics, the retention will be proportional to the abutment height and conicity [[5], [6], [7]]. In addition, the excess of non-removed cement can induce an inflammatory response in the peri-implant tissue, being a deleterious condition for osseointegration [10].

Several implant systems have been evaluated aiming to combine optimal mechanical, biological, and aesthetics of the prosthesis, reducing the above-mentioned disadvantages [11]. One of the previous purposed retention types was the lateral-screw-retained implant prosthesis in which an extra lateral screw is used to retain the crown instead of the conventional screw accessed through the occlusal surface [12]. This technique has been first purposed in 1995 [13], named as lingual locking screw. However, some synonymous can be found, such as Cross-pin retained implant-supported restoration [12] or Lateral-screw-retained implant prosthesis [14]. According to previous authors, this design allowed the implementation of properly shaped occlusal surfaces improving the esthetic of the prosthesis and reducing the occlusion interference [12,14]. Despite that, experimental and clinical studies with Lateral-screw-retained implant design showed insufficient proof of the treatment reliability. In addition, to allow the screw to tighten with adequate torque, it was recommended that the crown should present at least the lingual face in metal, without esthetic veneering, since the lateral screw required fixation in both crown and abutment [[11], [12], [13], [14], [15]].

Considered an evolution of Lateral-screw-retained implant prosthesis design, the Modified lateral-screw [14] or Anti-loosening inner-post screw [11] has been introduced. This design also uses a lateral metallic screw that is horizontally placed on the lingual face of the crown. The novelty of this technique is that the lateral screw should be tightened in an extension of the prosthetic screw instead of in the abutment surface and, that the crown should present threads in the access hole [11]. Few studies have evaluated this modified design, affirming that the prosthesis is maintained in position due to the pushing force of the lateral screw; while passive fit, easy removal, esthetic and functional occlusal surfaces will be maintained as a conventional design [11,14].

The mechanical benefits of using an extra component (lateral screw) in the prosthesis setup should be better addressed, demonstrating the reduction of stress magnitude and increasing the safety factor for fracture incidence. A previous finite element analysis reported that the conventional prosthetic transversal screw is an option to reduce the risk of mechanical complications, both in the prosthetic screw and in the abutment screw [15]. However, both models considered in the reported investigation presented a lateral screw, and the comparison with the conventional cement-retained and screw-retained designs was not performed [14,15]. Therefore, the present study aimed to assess and compare the stress distribution in four different crown retention types for single-crown implant designs (cement-retained, screw-retained, lateral-screw retained, and modified lateral-screw retained) during axial and non-axial loading. The null hypothesis was that there would be no difference in the stress concentration regardless of the setup design. The alternative hypothesis was that different loading incidences would exhibit different stress magnitudes.

## 2 Methods

**Fig. 1:**
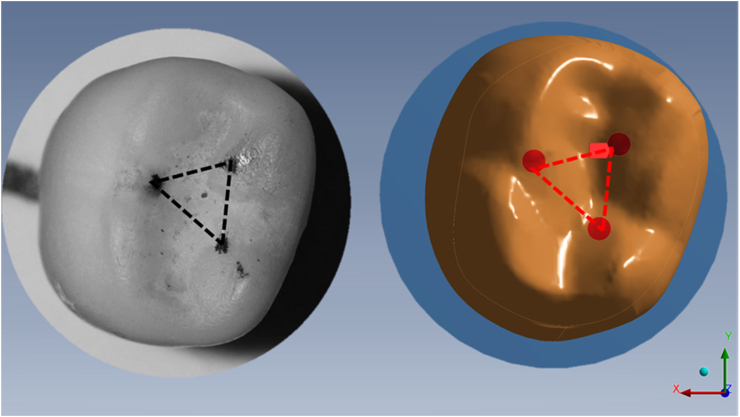
Loading region at the occlusal surface defined in the three-dimensional (3D) modeling software according to an in vitro measurement.

For the present study, a previous three-dimensional model of a dental implant was considered [16]. The dental implant model consisted of a fixture, a separate abutment, and its prosthetic screw. The initial geometries were based on the external design from a commercial implant system (Titaoss® TM cortical Intraoss®, SP, Brazil). Additionally, the crown was considered to present a 7.5 mm height from the abutment platform, 8.5 buccolingual, and 8 mm mesiodistal. For posterior contour definitions, a loading area was defined in the occlusal region of the crown, based on compressive loading setup (Fig. 1). A milled implant-supported crown was loaded with 600 N in the universal testing machine (Instron 6022, Instron Corp.) with articulating paper (Bausch articulating paper; Bausch Inc, Nashua, NH) and an occlusal photo was registered. Then circular areas have been defined according to the marks from it.

**Fig. 2:**
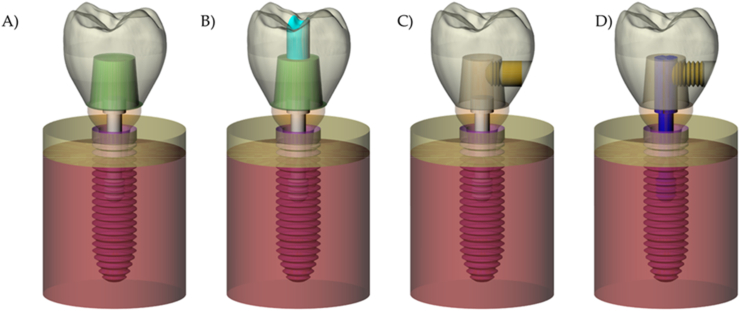
Numerical models used in the finite element analysis. Model A (cement-retained), Model B (screw-retained), Model C (lateral-screw-retained) and Model D (modified lateral-screw-retained).

Each 3D volumetric solid has been exported in Standard for the Exchange of Product Data (STEP) and modified into the computer-aided design software (Rhinoceros version 5.0 SR8, McNeel North America, Seattle, WA). To allow a similar number of faces between the abutment and the implant, and abutment and prosthetic screw, the Boolean difference was applied between them, allowing a perfect fit contact region. Then, the models were replicated in four different situations according to the crown retention system (Fig. 2A–D).

• Model A presented a cement-retained crown. In this model, a cement layer (in green) between the crown and abutment was considered [17].
• The model B presented a screw-retained crown. For that, a cement layer similar to the model was considered and the occlusal access hole was modeled from the extruded curve from the prosthetic screw head. In addition, a cylinder solid was modeled to simulate the resin composite sealing the screw access hole (in light blue) [16].
• The model C was created to simulate the lateral-screw-retained crown. For that, the lateral threads (in yellow) have been defined at the abutment side [13].
• The model D presented the modified lateral-screw-retained crown. For that, the central prosthetic screw (in dark blue) was modeled with a coronal extension, and the lateral screw presented threads (in yellow) in contact with the crown and the central screw [14].

**Fig. 3:**
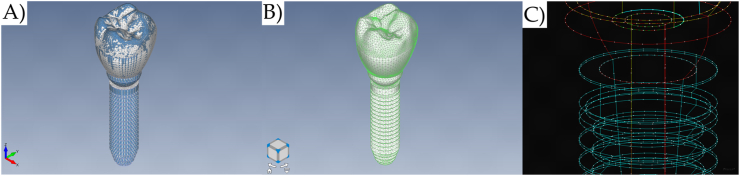
A) Meshing subdivision with superimposed solids, B) Nodes visible for the coincidence nodes verification, and C) Coincident curves used in the meshing refinement.

The Finite Element pre-processing was carried out using FEMAP 11.1.2 (Siemens PLM Software, Plano, Texas, USA), while the analysis was done with NX Nastran (Siemens PLM Software, Plano, Texas, USA). The mesh density was based on the individual meshing for each solid structure, considering a similar quantity of nodes between juxtaposed curves from contacting geometries (Fig. 3A–C). The mesh was manually checked for each model according to the feature “Model Data Contour” in the software. In addition, the Node Spacing was checked without biasing between the nodes transition in contacting geometries. The size of the element was 0.3 mm. For all models, the parabolic tetrahedron solid elements with mid-side nodes were used. After the process the models A, B, C and were composed respectively of 68,069, 100,530, 102,871, and 85,827 elements.

For the simulated linear elastic materials with isotropic behavior, the properties information were based on the literature. An elastic modulus of 107 GPa was used for titanium [18] and 210 GPa for the crown in Cobalt chromium [19], both with a Poisson ratio of 0.3. For the resin composite, the elastic modulus was 12.34 GPa [20] while the resin cement was simulated with 8.2 GPa [21]. The contacts were defined based on the coincident nodes, previously merged at the meshing process. To determine whether the structure is still in the elastic or plastic regime, after the post-processing, the Yield stress from each evaluated material was plotted in Table 1, according to the literature.

**Table 1:** Mechanical properties of the evaluated materials.

Material	Yield stress (MPa)	Reference
Titanium (Ti–6Al–4V)	850 to 1100	[22]
Cobalt chromium	875.42 to 893.33	[23]
Resin composite	107.20 ± 10.23	[20]
Resin cement	–	[21]

Following the delimited surface of the in vitro specimens, the Computer-Aided Design (CAD) model was adjusted to compose this area. Then, the load of 600 N was applied as a Total Load on Surfaces on the occlusal region delimited in the CAD (Fig. 1). This study assumed an average biting force of 600 N based on the data reported for molars under normal mastication [24]. A magnitude approach that was also previously simulated in other studies with 3D finite element analysis (FEA) and dental crowns [4,16,25,26].

**Fig. 4:**
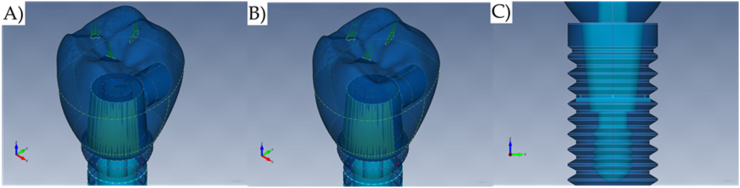
Boundary conditions. A) Axial loading, B) Oblique loading, and C) Fixed support.

Two different loading conditions were considered for each model (Fig. 4A and B). The first scenario was defined by the axial loading application, normal to the occlusal surface in three points of the masticatory surface of the tooth (FX = 0 N, FY = 0 N, FZ = 600 N) [4]. The second scenario was defined according to the International Organization for Standardization (ISO) 14,801:2016, in which the boundary condition should simulate the worst-case scenario with the implant's long axis at a 30° angle in the loading direction (FX = 0 N, FY = 300 N, FZ = 519 N). The nodes at the surface of the third thread of the implant fixture (Fig. 4C) have been selected as the constraints, restrained in all directions totaling 3 mm of exposition according to the ISO.

In the post-processing, the contour option “average elemental” without the use of the “corner data” was used for visualizing the results. The required results were in solid von-Mises stress.

## 3 Results

**Fig. 5:**
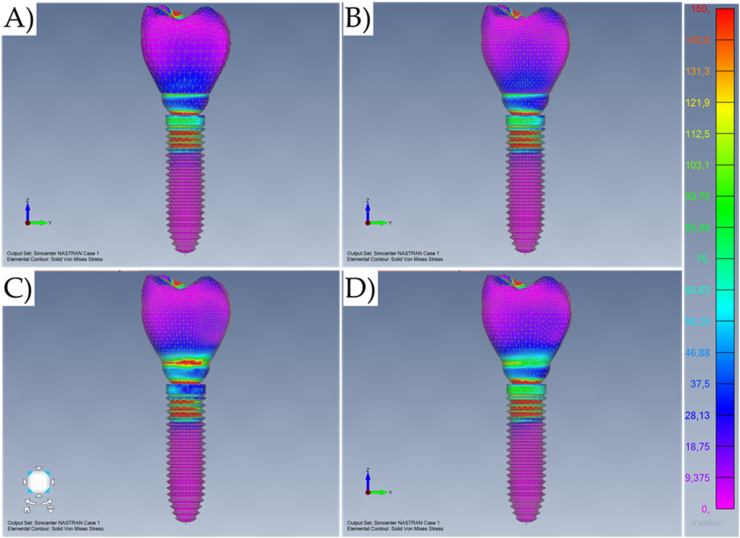
Von-Mises stress maps (MPa) in the external surface of the different systems during axial loading. A) Cement-retained. B) Screw-retained. C) Lateral-screw-retained. And D) Modified lateral-screw-retained.

**Fig. 6:**
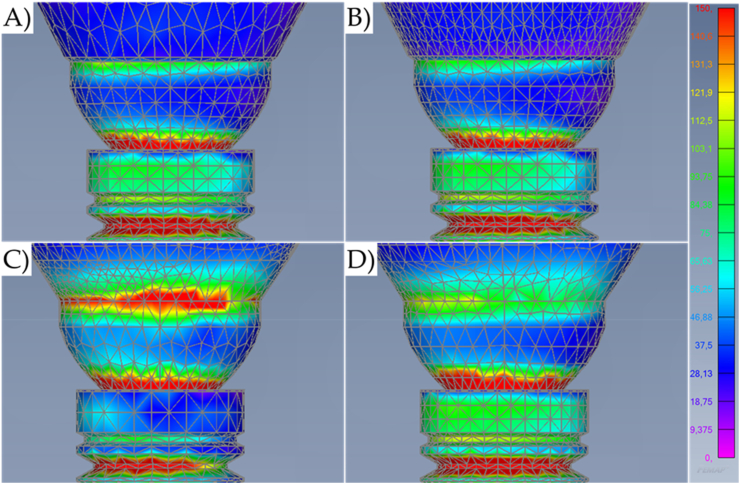
Von-Mises stress maps (MPa) in the external surface of the different systems during axial loading with a higher magnification at the cervical region. A) Cement-retained. B) Screw-retained. C) Lateral-screw-retained. And D) Modified lateral-screw-retained.

**Fig. 7:**
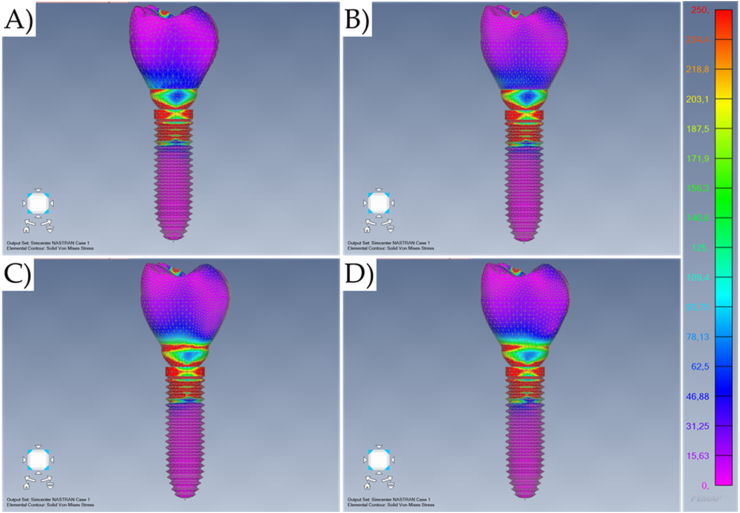
Von-Mises stress maps (MPa) in the external surface of the different systems during oblique loading. A) Cement-retained. B) Screw-retained. C) Lateral-screw-retained. And D) Modified lateral-screw-retained.

**Fig. 8:**
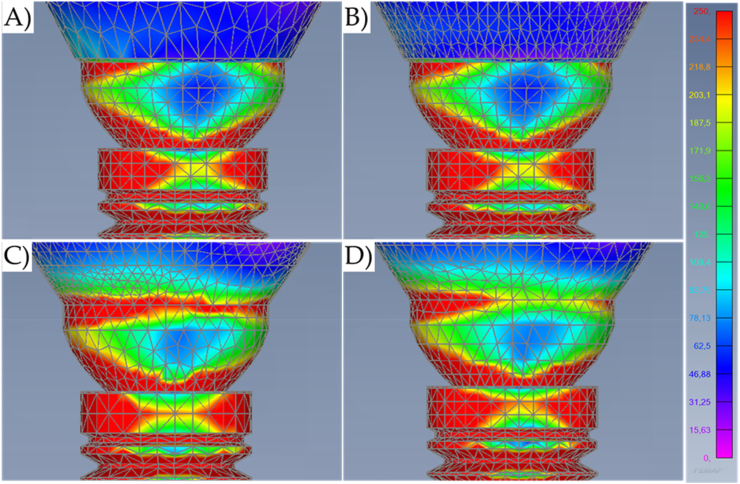
Von-Mises stress maps (MPa) in the external surface of the different systems during oblique loading with a higher magnification at the cervical region. A) Cement-retained. B) Screw-retained. C) Lateral-screw-retained. And D) Modified lateral-screw-retained.

The numerical analysis showed that different crown retention designs can modify the stress magnitude for unitary implant-supported restorations. The maximum stress (solid von-Mises stress) distribution can be observed in the models resulting from axial loading (Fig. 5, Fig. 6D) and oblique loading (Fig. 7, Fig. 8D).

**Fig. 9:**
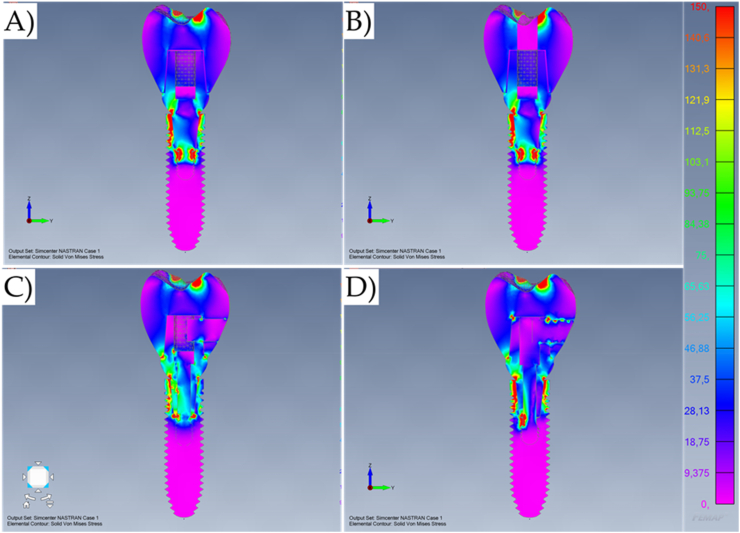
Von-Mises stress maps (MPa) in the section plane of the different systems during axial loading. A) Cement-retained. B) Screw-retained. C) Lateral-screw-retained. And D) Modified lateral-screw-retained.

**Fig. 10:**
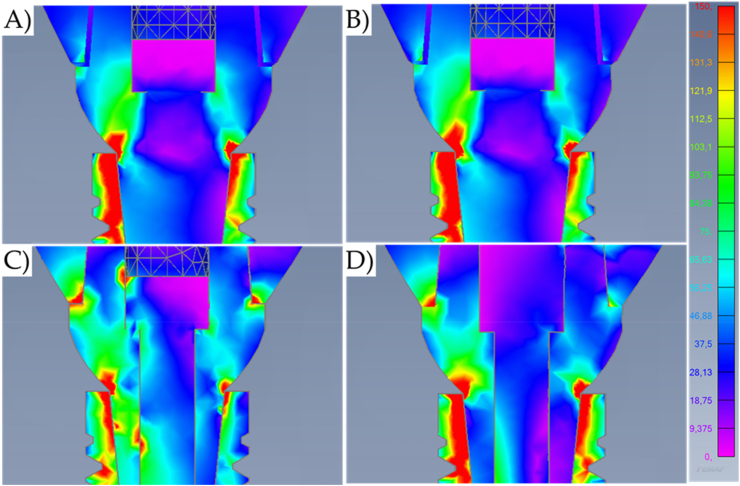
Von-Mises stress maps (MPa) in the section plane of the different systems during axial loading with a higher magnification at the cervical region. A) Cement-retained. B) Screw-retained. C) Lateral-screw-retained. And D) Modified lateral-screw-retained.

**Fig. 11:**
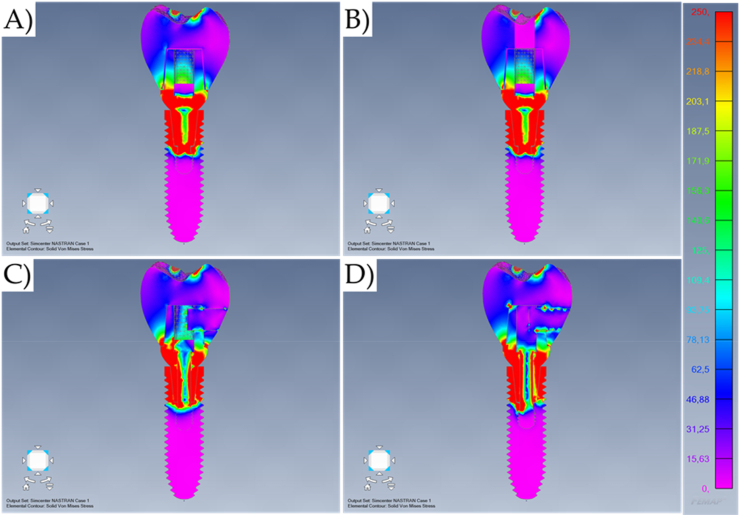
Von-Mises stress maps (MPa) in the section plane of the different systems during oblique loading. A) Cement-retained. B) Screw-retained. C) Lateral-screw-retained. And D) Modified lateral-screw-retained.

**Fig. 12:**
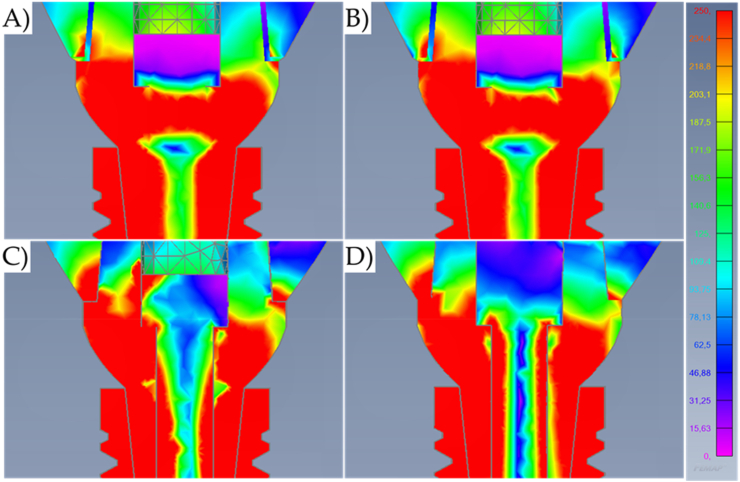
Von-Mises stress maps (MPa) in the section plane of the different systems during oblique loading with a higher magnification at the cervical region. A) Cement-retained. B) Screw-retained. C) Lateral-screw-retained. And D) Modified lateral-screw-retained.

To observe the inner path of the stress distribution, a new coordinate system was created based on the central axis of the implant model, and a section plane was performed on the buccolingual direction for axial (Fig. 9, Fig. 10D) and oblique loadings (Fig. 11, Fig. 12D).

Observing the stress distribution, there is a similar stress pattern between the models for the mechanical response in the implant threads; and regardless of the loading incidence and model design, the stress concentration occurred mainly in the cervical area of the geometry setup (Fig. 5). In addition, the stress did not concentrate on the bulk of the crowns. However, the models with a lateral fixation screw contacting the implant-abutment interface (Models C and D) presented the highest region of stress concentration at the crown margin (Fig. 6).

The similarities between cement-retained (Model A) and screw-retained (Model B) do not allow the assumption of crown design effect on the mechanical response for both models (Fig. 7, Fig. 8). However, it is visible that the cement-retained presents some stress around the occlusal resin composite suggesting that the adhesive interface could be compromised in long-term (Fig. 9).

Results show that the oblique loading direction as a non-axial vector force, highly increases the stress magnitude for all evaluated models (Fig. 11, Fig. 12). The difference caused by the crown retention type in the stress magnitude has been reduced with the incidence of the oblique loading but is still visible for Model C which showed the highest stress between the crown and the abutment joint (Fig. 8). In an approximate view of the stress map from the cervical region, with a section plane, it is possible to observe that Models A and B behave very similarly with reduced stress for the crown margin and higher stress for the prosthetic screw while Models C and D impaired the crown margin stress level and dampened the stress at the screw (Fig. 12). This effect was more notable in the model D, as a consequence of its lateral screw that was also presenting higher stress magnitude.

The results of the finite element calculations for the maximum von-Mises stress in the different regions of the specimen are summarized in Table 2. The region of force application was considered a singularity and not computed as a maximum stress peak.

**Table 2:** Stress peaks (MPa) calculated according to the loading incidence and model design.

Structure	Axial loading				Oblique loading			
	Model A	Model B	Model C	Model D	Model A	Model B	Model C	Model D
Crown	65.72	59.70	193.69	154.15	241.60	246.64	451.16	464.88
Region	Finishing line				Finishing line			
Abutment	196.85	207.51	180.04	176.87	830.69	855.36	1023.02	1011.91
Region	Abutment neck				Abutment neck			
Screw	119.28	117.43	120.16	148.15	476.13	444.79	301.15	288.25
Region	First thread				Screw neck			
Implant	263.24	260.32	183.07	201.96	974.52	1007.75	1028.36	1097.38
Region	The internal wall of the connection				The external wall at the third thread			
Lateral screw	–	–	106.27	155.08	–	–	146.25	234.40
Region	–	–	Contact with the abutment	Contact with the crown	–	–	Contact with the abutment	Contact with the crown

For the vertical prosthetic screw, the highest stress magnitude was approximately 26% higher for model D than model B, during axial loading. When comparing both models with lateral screws (C and D), the difference was also present; however, in a smaller proportion (23%) with the highest magnitude for the model D that have the modified prosthetic screw. However, for the oblique loading, model D showed an opposite behavior, with a lower stress magnitude for the prosthetic screw than model C (4.47%). In the simulated conditions, the stress maps indicated that part of the stress was concentrated at the lateral screw neck (Model C with higher stress with axial loading and Model D with higher stress with oblique loading), suggesting a new possible failure origin for these models and consequently a reduced failure risk for the vertical prosthetic screw in comparison with model A and B.

**Fig. 13:**
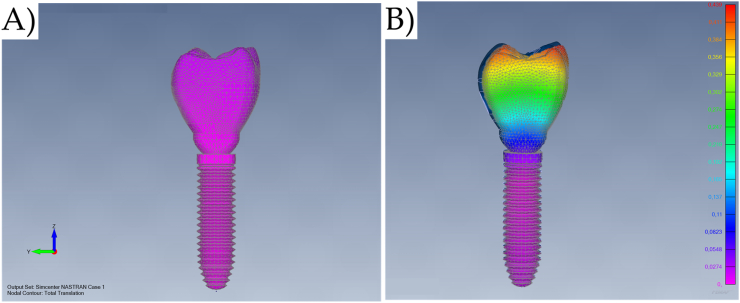
Representative total translation (mm) with Modified lateral-screw-retained crown designs with A) Axial and B) Oblique loading.

During the incidence of axial occlusal loadings in an anatomic crown, it can be still expected a non-axial component due to the slope of the contact points. This loading variation is predominant due to the shape of the geometry, and in this case due to the simulation of complex 3D geometries that are not radially symmetrical. Since the occlusal shape was identical for all models, the maximum displacement for axial loading was 0.02 mm regardless of the model design. However, the incidence of the oblique loading method, defined by decomposing the vectorial components instead of a uniaxial definition was more capable of bending the set (0.46 mm) regardless of the prosthetic crown design. Fig. 13 summarizes the total translation in mm when the different loads were applied in the same model.

## 4 Discussion

The present investigation showed that the stress distribution of implant-supported posterior restorations was affected by the crown retention system and loading direction. According to Tables 1 and it was evident that the simulated conditions of the dental implant generated an effect on the stress peaks. The null hypothesis was therefore rejected. However, the results showed that different loading directions could affect the treatment biomechanics. Therefore, the alternative hypothesis has been accepted.

A previous finite element study assessed the stress distribution in the screws and abutment of a single-crown implant with transversal or transocclusal screw models. The authors found that the stress was higher with more oblique loads when the transversal screw model was simulated. In a simplified explanation, the model named as transversal screw model could be compared to the present model C and the transocclusal group could be compared to the present model B. Therefore, the present study corroborates with their results indicating a higher stress magnitude in the lateral screw when oblique loading was considered. However, according to the previously reported study, the prosthetic lateral screw showed less stress than the rest of the components being the structure with the lowest risk of mechanical complications. In this statement, the present results diverge from them since it is possible to see that the presence of lateral screws (models C and D) can damp the stress in some structures, however increasing the stress on its structure, an effect that cannot be assumed as the option with the lowest failure risk. These differences between the previous study and the present results should be inherent to the model design, contact conditions, meshing method, and different structures that were present in this model but not in the previous investigation (such as the cement layer and anatomic crown) [15].

The incidence of perpendicular loads to biomedical titanium implants is widely investigated in the field of orthopedics. A finite element investigation showed that oblique loading increased the von-Mises stress peak in the fixation models and concentrated more stress at the additional middle screw of titanium implants used for the treatment of ulnar head fracture [27]. This behavior according to the authors, can be justified by the increased bending moment that this screw was submitted during loading. Even for dental implants, the two most probable causes of abutment screw loosening are excessive bending of the screw joint and settling effects. In the case of increased bending moment, when the load generated is larger than the yield strength of the screw, plastic deformation will occur, reducing the contact between the abutment and the screw, making the restoration loose [28]. However, for the titanium alloy (Ti6Al4V), the Yield stress is approximately 850 MPa [29], which means that with 600 N in the simulated condition, the implants fixture would fail before the prosthetic screws, under oblique load. According to the Yield stress values (Table 1), no plastic deformation is expected in the crown and the lateral screw, regardless of the design or loading mode. However, for the abutment, models B, C, and D are within the range of possible damage when an oblique load is applied.

According to a critical review, in screw-retained implant-supported restorations, the fastening screw can provide a solid joint between restoration, abutment and implant. While in cement-retained prostheses, this restorative screw is eliminated for esthetics, occlusal stability, and improved passive fitting. In addition, the presence of an intermediate cement layer can act as a shock absorber and enhance the transfer of load throughout the prosthesis-implant-bone system [7]. In the present study, however, the simulated prosthetic piece was a conventional abutment and not a universal castable long abutment, therefore the cement layer was present for both models A and B. Despite that, the difference between both models is too small to assume any significant effect on stress clinically relevant.

Based on a random-effects Poisson regression analysis, a systematic review determined that the estimated 5-year survival rate of screw-retained is similar to that for cemented reconstructions (96.03%). However, the fracture of ceramic was significantly more frequent in screw-retained systems compared to the cemented ones. The loosening of the abutment was more frequent with cemented crowns [6]. According to the literature, the mechanical stability of the prosthetic components in the implant-prosthesis complex is essential to the long-term success of the restorations. Comparing screw- and cement-retained implant-supported prostheses by using the finite element method, another investigation reported that screw-retained prostheses showed a higher risk of screw loosening and fracture [8]. According to the authors, implant-supported fixed partial prostheses with 3 elements can show a higher magnitude of von Mises equivalent stresses as well as gap formation [8]. This effect was not observed in the present study, with similar mechanical behavior for both conditions, corroborating with previous report [6].

A retrospective clinical study evaluated complications for dental implant-supported restorations with different prosthetic designs and observed that peri-implant mucositis was most frequently observed in the Anti-loosening inner-post screw (Current model D) prosthesis design (21.9%). However, the authors affirmed that the biological complications were not affected by the retention type [11]. In addition, it was reported 43.8% of screw loosening for the same Anti-loosening inner-post screw prosthesis design [11]. The reported failures can be explained by the high-stress magnitude generated in the lateral screw during the axial or oblique loading observed in the present study.

Another clinical trial evaluated only the Anti-loosening inner-post screw prosthesis design rehabilitated patients and concluded that the maintenance care facilitates the prevention of mechanical and biological complications with this alternative design [14]. However, the authors did not present another prosthesis design to compare the results. Therefore, more studies are necessary to corroborate or not with these findings.

When first introduced, the concept of a secondary lateral screw was created to allow a prosthetic design with good aesthetics, and retrievability [13]. Later, the technique was reintroduced with the name of cross-pinning implant-supported prostheses [30] and was recommended when an abutment screw's access hole would compromise the aesthetics and/or structural integrity of the prosthesis [30]. The disadvantages of the lateral screw include increased cost for restoration construction, increased technical complexity (at a laboratory level), and biological complications related to leakage [12]. The same authors performed an in vitro study and showed that cement gaskets may effectively prevent leakage into the lateral screw. However, the use of cement as a gasket reduces the predictable retrieval and can promotes cement extrusion and finally incomplete seating [12].

The lateral screw design was also reported for zirconia frameworks. A case report presents an alternative technique involving prostheses made by 3 mol% yttria-stabilized tetragonal zirconia polycrystal (3YTZP) attached to custom abutments using transverse screws. According to the authors, this technique seems to be a viable alternative to traditional metal-ceramic screw-retained restorations when retrievability and esthetics are desired parameters for restoration success [31]. However, there is no report of in vitro performance or clinical data to allow a direct extrapolation.

In the present study, all simulated materials have been considered isotropic. FEA studies of dental materials often use isotropic material models as a point of reference for comparison. Analyzing the behavior of isotropic materials can provide researchers with valuable insights into the expected performance of dental materials under varying loading conditions. Therefore, results obtained from the numerical model can then be compared with those generated by previous studies, allowing researchers to assess the degree of reliability and refine their analyses accordingly. Despite being considered a sort of simplification, FEA isotropic models can be used as a valid approach for analyzing dental materials, particularly when anisotropic models are not available or when the level of anisotropy is low [[32], [33], [34], [35], [36]]. In summary, the stress results assist in overcoming the challenges involved in accurately modeling the behavior of dental materials that can present complex properties [37].

Considering the adopted analysis criterion, the von-Mises stress was chosen due to its common application in FEA to evaluate the safety and reliability of mechanical structures and components. It provides a measure of the stress level that a material experiences, taking into account all of the different types of stress (e.g., tensile, compressive, and shear) that are present. The von-Mises stress is particularly useful when analyzing materials that exhibit plastic deformation, as it can predict the onset of yielding and failure more accurately than other stress measures when considering metallic dental implants [38,39].

A previous study found that higher stress levels were observed under buccolingual loading compared to axial loading when loading implants. The study highlighted the significance of progressive marginal bone loss and excessive loading, indicating that they can lead to higher stresses in the bone tissue, potentially affecting the bone remodeling process [40]. In this scenario, considering that these factors can influence bone remodeling, further studies addressing their implications on bone health and tissue integrity would contribute significantly to our understanding of implant biomechanics [40,41].

As limitations of a finite element analysis, this investigation did not simulate humidity, chewing, pH, and temperature variations that are present in the human mouth dynamic medium. The models were simulated only for posterior crowns, with perfect fitting and absence of defects incorporation. In addition, other studies simulating different restorations and bone conditions should be evaluated to corroborate the present findings. It's essential to emphasize that finite element simulation serves as a valuable tool for predicting specific behaviors in controlled scenarios. This capability can prove highly effective in guiding future treatment decisions [42]. Based on the findings of this study, future research in implant-supported restorations should emphasize longitudinal clinical studies to evaluate the longevity and performance of various crown retention systems. Additionally, there is a need for investigations into the incorporation of novel materials and alloys in the design of these retention systems.

## 5 Conclusion

Based on this limited investigation, the different crown retention systems can modify the stress concentration during loading incidence in implant-supported restorations. The use of retention systems with an extra horizontal screw can reduce the stress generated at the prosthetic screw and implant, however, it increases the stress magnitude at the crown as well as in the lateral screw itself. This effect is attenuated during non-axial loading when all the evaluated models showed a more prone failure condition at the cervical level, regardless of the prosthesis design.

## Ethical approval

Not required.

## Data availability statement

The data that support the findings of this study are available from the corresponding author upon reasonable request.

## Availability of data and material

All data will be fully available after the request.

## Funding support

This research did not receive any specific grant from funding agencies in the public, commercial, or not-for-profit sectors.

## CRediT authorship contribution statement

**João Paulo M. Tribst:** Writing – review & editing, Writing – original draft, Visualization, Supervision, Project administration, Methodology, Investigation, Formal analysis, Data curation, Conceptualization. **Niek de Jager:** Writing – review & editing, Visualization, Validation, Supervision, Software, Resources, Project administration, Methodology, Investigation, Formal analysis, Conceptualization. **Amanda M.O. Dal Piva:** Writing – review & editing, Writing – original draft, Visualization, Validation, Conceptualization. **Cees J. Kleverlaan:** Writing – review & editing, Supervision, Project administration, Investigation, Conceptualization. **Albert Feilzer:** Writing – review & editing, Writing – original draft, Validation, Supervision, Software, Project administration, Investigation, Formal analysis, Conceptualization.

## Declaration of competing interest

The authors declare that they have no known competing financial interests or personal relationships that could have appeared to influence the work reported in this paper.
